# Newspapers, Medicine, and Charity

**Published:** 1899-12

**Authors:** 


### NEWSPAPERS, MEDICINE, AND CHARITY.

THE *New York Herald* sent a quantity of the Saranelli yellow fever serum to Havana a while ago and requested that it be given a trial by the surgeon of the Marine Hospital Corps. Naturally, under the circumstances, one must look for the first report of its use in the columns of the newspaper making the gift of serum. Such a report has been received by the *Herald*, from an Havana correspondent, and printed. It is in no sense a scientific report of results; merely a statement in narrative form. This is published elsewhere for what it is worth, and while, to scientific men, a recognised report of results published over a recognised name in a scientific journal would be more satisfactory, yet the correspondent's report of the trial is interesting.

Newspapers are into everything these days. By some this move of the *Herald* may be characterised as a stroke of the "yellow" enterprise, which has become so obnoxious to thinking, serious-minded people within the past few years. If it is so classified, then it is "yellow" in the right direction, for while the movement was inaugurated for advertising purposes, such plans might result in untold good, and it is a spending of money in the right direction. Just as *Life* and the *Tribune* advertising schemes, the fresh-air fund, has done untold good to hundreds of tenement tortured babies; and the *World's* free ice fund, and the *Journal's* free coal and free bread distribution. These are pure advertising schemes, but they carry with them much good to a great number of persons, and while to some ultra philanthropists, who prefer to investigate before relieving, it may seem cruel to use such methods for advertising purposes, the fact remains, nevertheless, that these newspapers do more good to the army of poverty-stricken sufferers, than all other methods combined.

Buffalo had an illustration of this a few years ago, during the "hard times" period, when want stalked forth and invaded the ragged homes of the unemployed. Organised charity investigated, and while its agents questioned and advised, the *Evening News* appealed to its readers for supplies. Food and clothing were asked for, and such a scene was enacted as was never before witnessed in Buffalo. Train load after train load of vegetables was sent into Buffalo in response to the appeal. Headquarters were established and bales of clothing and tons of food were distributed. Organised charity said the *News* was making paupers and putting a premium on poverty. Maybe so. But organised charity did not see the hollow-eyed, half-starved applicants who, while waiting relief, hungrily and publicly grabbed up raw potatoes or raw turnips and ate them before the eyes of the world in a public street of a city of over 300,000 inhabitants. That was plain hunger—the kind of hunger that means want and needs no investigation. That relief-plan advertised the *News*, but it filled many an empty stomach and clothed many a naked form, and the vast good done by the newspaper overshadowed the advertising it received. If advertising of this nature does newspapers any good then they are entitled to it, for the benefits and relief they confer are of far greater value than those which they receive.

